# Inhibition of the mevalonate pathway augments the activity of pitavastatin against ovarian cancer cells

**DOI:** 10.1038/s41598-017-08649-9

**Published:** 2017-08-14

**Authors:** Marwan Ibrahim Abdullah, Mohammed Najim Abed, Alan Richardson

**Affiliations:** 1 Institute for Science and Technology in Medicine, Guy Hilton Research Centre, Keele University, Thornborrow Drive, Stoke-on-Trent, UK; 2 School of Pharmacy, Keele University, Keele, United Kingdom

## Abstract

Only 40% of patients with advanced ovarian cancer survive more than 5 years. We have previously shown that pitavastatin induces regression of ovarian cancer xenografts in mice. To evaluate whether the response of ovarian cancer cells to pitavastatin is potentiated by farnesyl diphosphate synthase inhibitors or geranylgeraniol transferase I inhibitors, we evaluated combinations of pitavastatin with zoledronic acid, risedronate and GGTI-2133 in a panel of ovarian cancer cells. Pitavastatin (IC_50_ = 0.6–14 μM), zoledronic acid (IC_50_ = 21–57 μM), risedronate (IC_50_ > 100 μM) or GGTI-2133 (IC_50_ > 25 μM) inhibited the growth of ovarian cancer cell cultures. Combinations of pitavastatin with zoledronic acid displayed additive or synergistic effects in cell growth assays in 10 of 11 cell lines evaluated as well as in trypan blue exclusion, cellular ATP or caspase 3/7, 8 and 9 assays. Pitavastatin reduced levels of GGT-IIβ and the membrane localization of several small GTPases and this was potentiated by zoledronic acid. siRNA to GGT-Iβ and GGT-IIβ used in combination, but not when used individually, significantly increased the sensitivity of cells to pitavastatin. These data suggest that zoledronic acid, a drug already in clinical use, may be usefully combined with pitavastatin in the treatment of ovarian cancer.

## Introduction

Ovarian cancer is the 5^th^ leading cause of death in women with more than 14,000 deaths reported annually in United States^1^. The disease responds initially to treatment which is most often surgical cytoreduction followed by chemotherapy^2^. The primary response rates to chemotherapy are approximately 80%^3^. Unfortunately, most patients relapse after a period of remission^4^ and eventually tumors becomes refractory to frontline therapy^2^. The lack of widely effective therapies at this point leads to a low 5-year survival of approximately 40%^3, 5^. Therefore, new therapeutic agents or treatment strategies are required.

The mevalonate biosynthetic pathway is responsible for the synthesis of several important metabolites, producing cholesterol, dolichol, ubiquinone and the isoprenoids farnesol and geranylgeraniol. The rate limiting step in the mevalonate pathway is hydroxymethylglutaryl coenzyme A reductase (HMGCR) which catalyses the production of mevalonate. HMGCR is expressed in clinical samples of ovarian cancer^6^ and HMGCR has been identified as metabolic oncogene which promotes xenograft growth and co-operates with Ras^7^. HMGCR activity may be deregulated in tumours, becoming resistant to negative feedback control by sterols and this may help provide an abundance of isoprenoids to promote growth of transformed cells^8^. These isoprenoids are used to post-translationally modify several small GTPases superfamily proteins and support their membrane localization^9^. Many members of the small GTPase family are oncoproteins and play critical roles in human oncogenesis^10^. Three prenyl transferase enzymes are known to catalyse the addition of isoprenoids to small GTPases. Geranylgeranyl transferase I (GGT-I) catalyses the geranylgeranylation of Rho family proteins while geranylgeranyl transferase II (GGT-II) performs the geranylgeranylation of the Rab protein family. Farnesyltransferase (FTase) is responsible for the farnesylation of Ras family protein. Prenyl transferase enzymes may also be deregulated in cancer. For example, geranylgeranyl transferase-β enzymes had been reported to be upregulated in several human tumors^11^. Collectively, this has raised interest in the mevalonate pathway as a potential target in oncology.

Statins are drugs which inhibit HMGCR and several studies have demonstrated that statins inhibit growth and induce apoptosis *in vitro* in cell lines from a range of cancer types^12–14^. Several studies have also reported that statins inhibit tumour xenograft growth in mice^15, 16^ and we have demonstrated that pitavastatin causes tumour regression in mice fed a controlled diet^17^. Epidemiological studies have found a reduced cancer risk and cancer related mortality in patients using statins for reduction of elevated cholesterol level (reviewed in ref. 18). Several, but not all, studies have found improved survival of ovarian cancer patients who are also statin users (reviewed in ref. 19). We, and others, have shown that relatively high doses of statins are likely to be necessary to achieve an adequate plasma concentration of drug^20, 21^. However, this raises concerns about the potential risk of myopathy, a side effect commonly associated with statins. This makes it desirable to identify drugs which synergize with statins and potentially reduce the dose of statin that is necessary to treat patients.

Bisphosphonates (e.g. zoledronic acid, risedronate) are drugs which are already approved for the management and prevention of bone disease and bone metastasis^22^. Bisphosphonates can also inhibit the mevalonate pathway enzyme farnesyl diphosphate synthase^23^. Inhibition of farnesyl diphosphate synthase depletes both farnesyl diphosphate and geranylgeranyl diphosphate which in turn are required for isoprenylation of small G-proteins^24^. Bisphosphonates have shown potential anti-cancer activity in different cancer cell lines including ovarian, colon and hepatic cells (reviewed in ref. 22). In addition, several studies showed that bisphosphonate use correlates with reduced cancer risk^25, 26^. Bisphosphonates can also enhance the anticancer activity of several chemotherapeutic agents *in vitro* ^27–30^.

Recent results from our laboratory have suggested that pitavastatin is superior to other statins for use in oncology because it is the only statin that is both lipophilic, rendering it more potent than hydrophilic statins, and has a suitably long half-life (t_1/2_ ~11 hr)^17, 31–33^. The latter property is important because we have shown continual inhibition of HMGCR is necessary to induce cell death and the troughs in plasma drug concentration between prolonged dosing intervals using short half-life statins are likely to compromise the activity of statins^33^. To reduce the dose of pitavastatin necessary in patients, and potentially minimize adverse effects, we have investigated whether zoledronic acid, risedronate or the geranylgeranyl transferase I inhibitor, GGTI-2133 potentiate the activity of pitavastatin. We show that zoledronic acid displays synergistic effects with pitavastatin in most of the ovarian cancer cell lines tested. We also evaluated the contribution of prenyl transferases to the activity of pitavastatin. Inhibition of these enzymes individually does not potentiate the activity of pitavastatin but knockdown of both GGT-Iβ and GGT-IIβ simultaneously augments the activity of pitavastatin. These data can help guide future clinical trials and drug discovery programmes to identify drugs which can be combined with pitavastatin to treat ovarian cancer.

## Results

### Pitavastatin, zoledronic acid, risedronate and GGTI-2133 inhibit the growth of ovarian cancer cell lines

The growth inhibitory activities of pitavastatin, zoledronic acid, risedronate and GGTI-2133 as single agents were first determined against a panel of ovarian cancer cell lines. Pitavastatin displayed concentration-dependant growth inhibition with IC_50_s ranging from 0.67–14 µM (Table 1). Zoledronic acid had lower potency than pitavastatin but also showed concentration-dependent growth inhibitory activity with IC_50_s ranging from 21–60 µM (Table 1). In contrast, risedronate (IC_50s_ > 100 µM) and GGTI-2133 (IC_50_ > 25 µM) did not show significant activity against ovarian cancer cell lines at the concentrations tested.

**Table 1:** Single agent potency of pitavastatin and zoledronic acid in cell growth assays.

IC_50_ (µM)		
Cell line	Pitavastatin (n)	Zoledronic acid (n)
HOE	0.69 ± 0.12 (6)	57 ± 6 (5)
A2780	0.67 ± 0.34 (9)	29 ± 4 (4)
CisA2780	14.0 ± 7.00 (9)	36 ± 6 (8)
Cov-318	3.40 ± 1.40 (8)	28 ± 2 (4)
Cov-362	3.10 ± 0.70 (8)	42 ± 4 (4)
Ovcar-3	4.60 ± 0.90 (6)	60 ± 4 (6)
Ovcar-4	5.20 ± 1.20 (4)	51 ± 7 (4)
Ovcar-5	2.40 ± 1.30 (9)	30 ± 6 (9)
Ovcar-8	0.40 ± 0.10 (4)	21 ± 3 (4)
Igrov-1	1.60 ± 0.10 (9)	43 ± 8 (7)
Skov-3	3.60 ± 1.00 (5)	26 ± 5 (5)
Ovsaho	0.69 ± 0.12 (5)	44 ± 7 (3)

Cells were exposed to a range of concentrations of pitavastatin or zoledronic acid for 72 hr, except for the slow growing cell lines Cov-318 and Cov-362 (120 hr). The numbers of surviving cells were estimated by statin with SRB. IC_50_s (mean ± S.D.) were calculated from the indicated number (n) of experiments.

### Pitavastatin and zoledronic acid synergistically inhibit the growth of ovarian cancer cell lines

**Figure 1:**
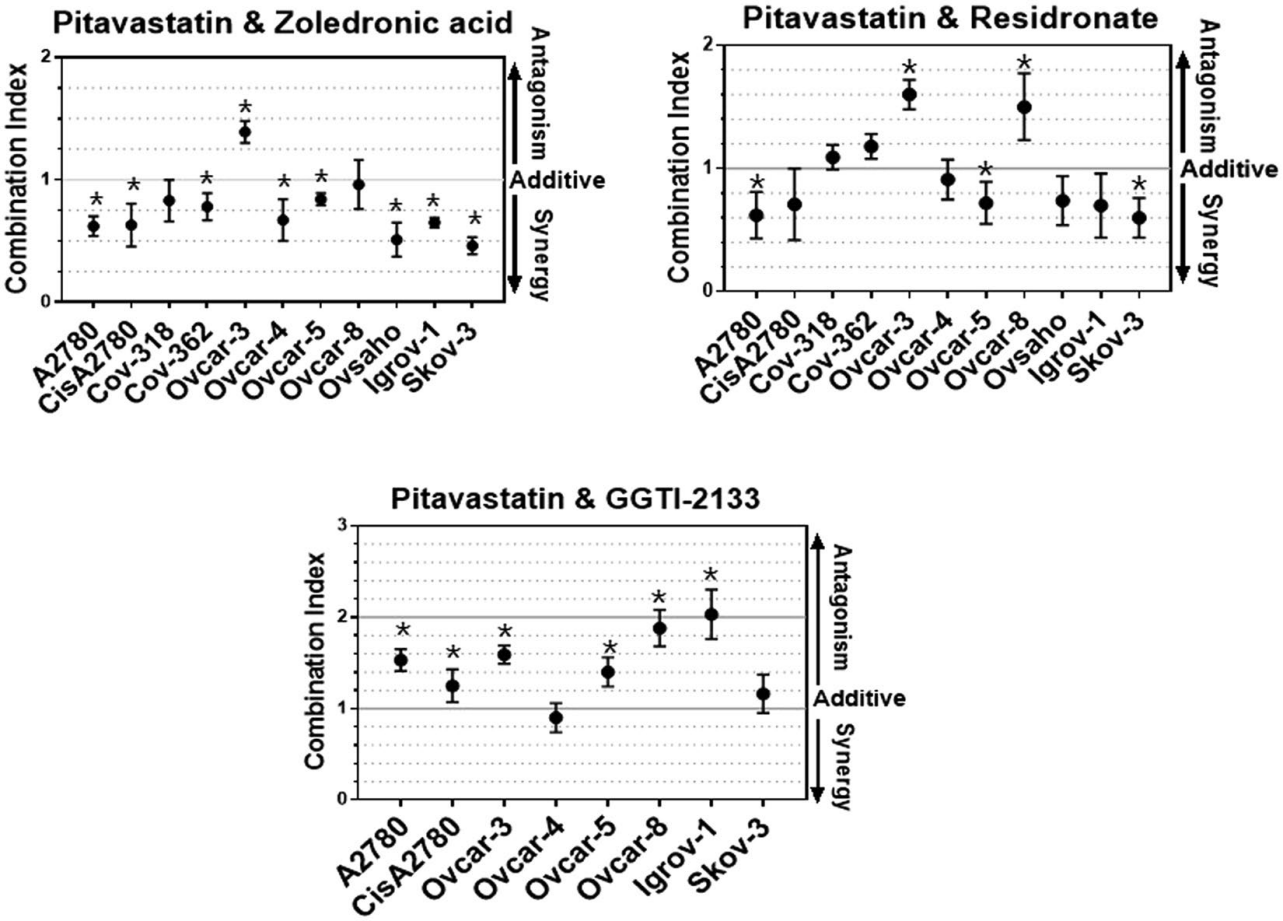
The effect of pitavastatin combinations in cell growth assays. To measure the activity of pitavastatin in combination with other agents, the indicated cells were simultaneously exposed to a range of pitavastatin concentrations with fixed concentration of zoledronic acid (10 µM), or risedronate (10 µM) or GGTI-2133 (5 µM). Combination indices (CI) (mean ± S.D., n = 3–4) are quoted at a fraction affected of 0.5 and differed significantly from unity where indicated (*Paired t-test, P ≤ 0.05).

The lack of potent activity of the bisphosphonates and GGTI-2133 led us to evaluate these drugs at fixed concentrations in combination with a range of concentrations of pitavastatin in cell growth assays (recommended in ref. 34). Pitavastatin and zoledronic acid displayed synergistic activity in 8 of 11 cell lines tested, additive activity was observed in two cells and antagonism was observed in one cell line (Ovcar-3 cells). When pitavastatin was combined with risedronate, synergy was observed in 3 cell lines, additivity in 6 cell lines and an antagonistic interaction was observed in two cell lines (Ovcar-3 and Ovcar-8). In contrast, most of the cell lines showed an antagonist interaction when GGTI-2133 was combined with pitavastatin (Fig. 1). These data led us to focus on combinations of pitavastatin and zoledronic acid.

### Confirmation of the synergistic effect of the pitavastatin and zoledronic acid combination

**Figure 2:**
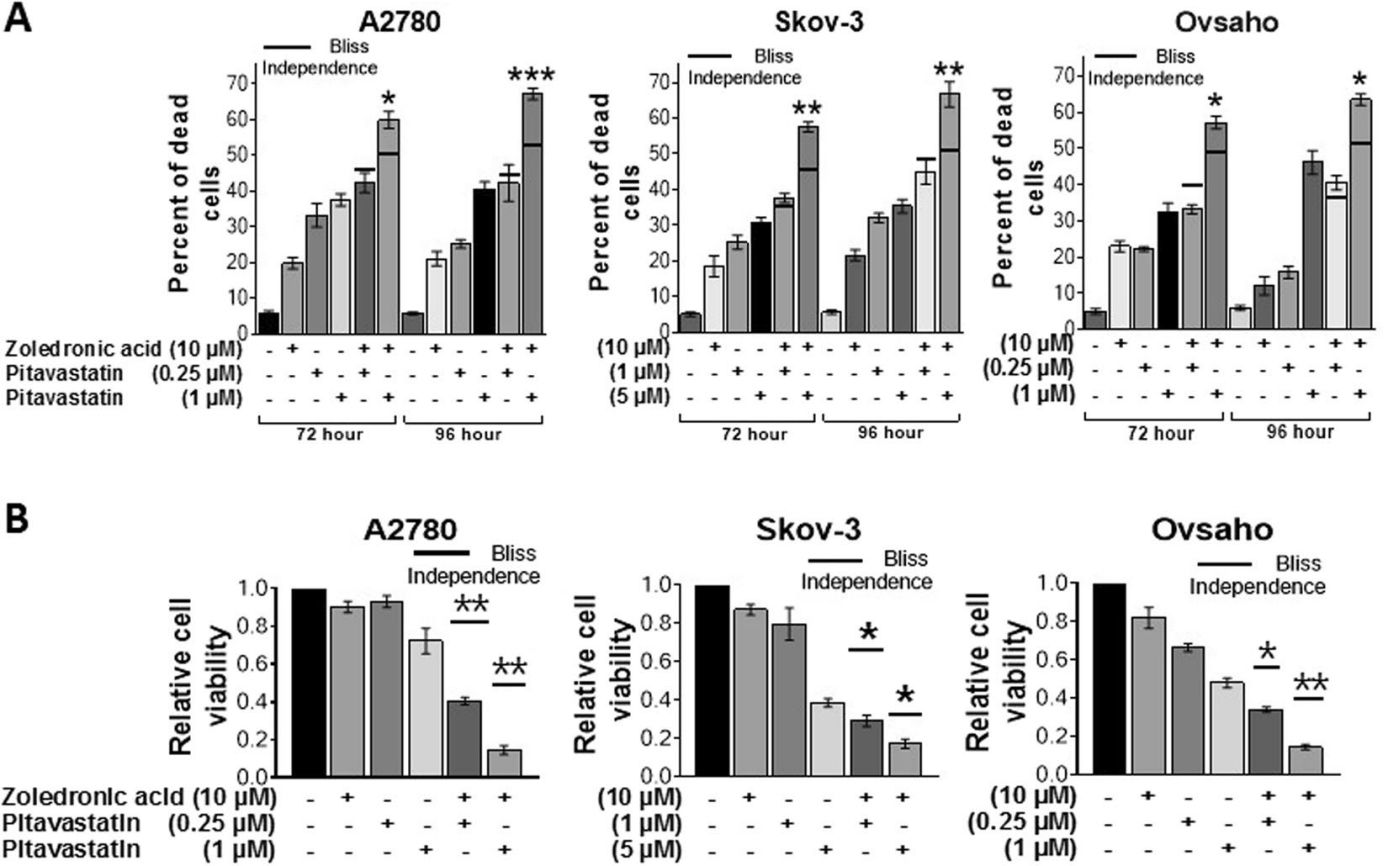
The effect of pitavastatin-zoledronic acid combinations on cell death. (**A**) Dead cells were measured by trypan blue staining after 72 and 96 hr of exposure to the indicated drug concentration. (**B**) Relative cell viability was measured by celltiter-Glo assay (ATP) after 72 hrs exposure to the indicated drug concentration. In both assays, the results (mean ± SD; n = 3) were compared to the effect expected for an additive interaction calculated using the Bliss independence criterion (solid line for each drug combination) and determined using the measured effect of the individual drugs in each individual experiment. Results were significantly different from the expected Bliss effect where shown (*P < 0.05; ** < 0.01; *** < *0.001*, paired t-test).

In order to confirm the synergy observed between pitavastatin and zoledronic acid, we choose A2780 (wild-type TP53), Skov-3 (deleted TP53) and Ovsaho (mutated TP53) cell lines because the most significant synergy had been observed in these cell lines and they differ in their *TP53* status. Cell death was first assessed by staining with trypan blue. The combination of pitavastatin with zoledronic acid resulted, in all three cell lines, in significantly more cell death after 72 and 96 hr of drug exposure than would have been expected from an additive effect calculated using the Bliss independence criterion (Fig. 2A). To confirm these results, we used a separate measurement of cell viability by measuring intracellular ATP level. After 72 hr of drug exposure, significantly less ATP was measured in cells exposed to the drug combination than that expected effect from an additive effect calculated using the Bliss independence criterion (Fig. 2B).

### Pitavastatin and zoledronic acid combinations synergistically induce apoptosis

**Figure 3:**
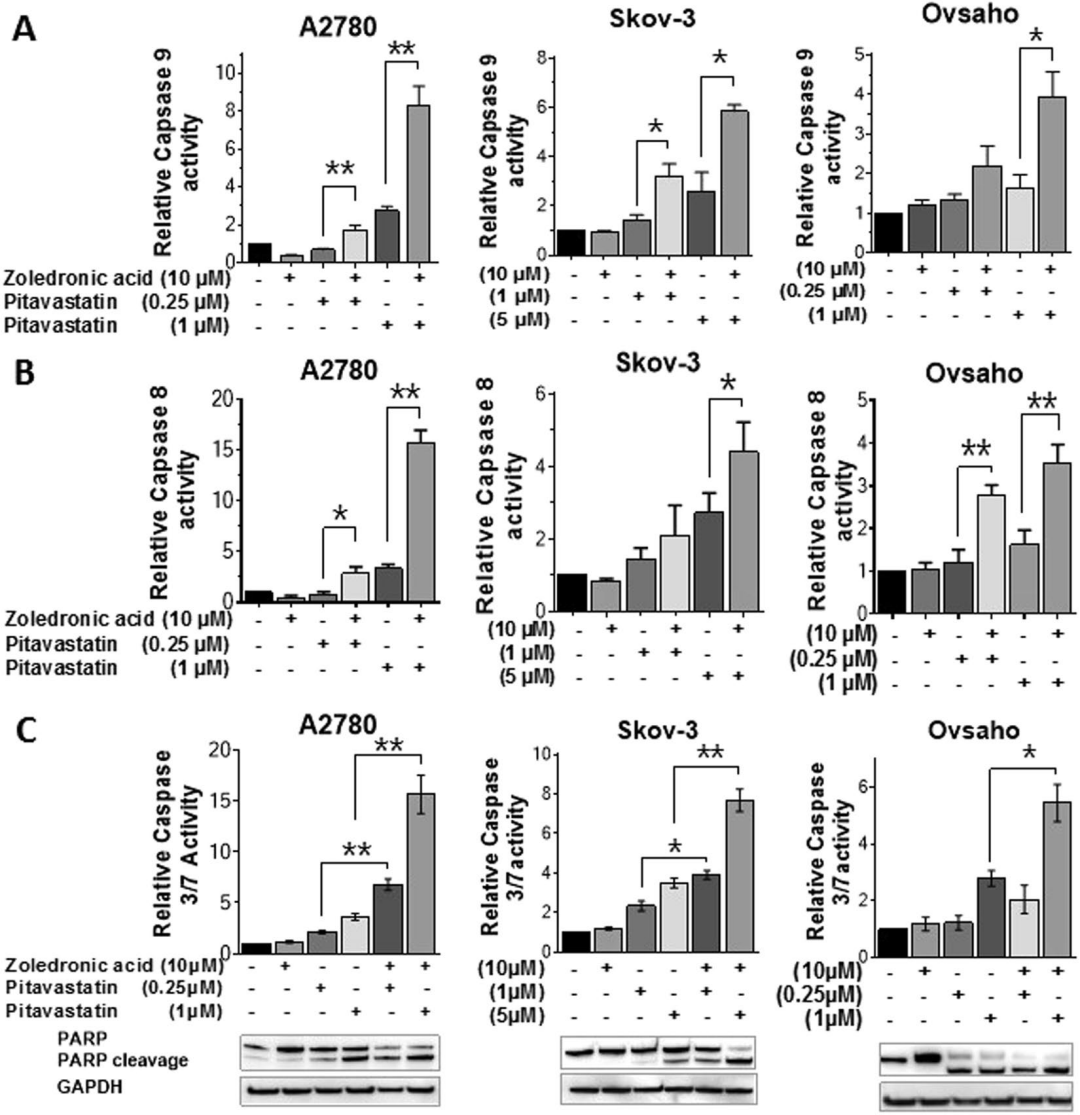
The effect of pitavastatin-zoledronic acid combinations on caspase activity. Caspase 8 (**A**), 9 (**B**), and 3/7 (**C**) activity of A2780, Skov-3 and Ovsaho cell lines were measured by Caspase-Glo assays. Cells were treated with the indicated concentrations of pitavastatin and zoledronic acid for 48 hr. The effect of the drug combinations were significantly different to the effect of pitavastatin alone where shown (mean ± SD; N = 3; *P < 0.05, ** < 0.01; paired t-test). PARP and PARP cleavage (**C**) were measured by western blot analysis (n = 3).

**Figure 4:**
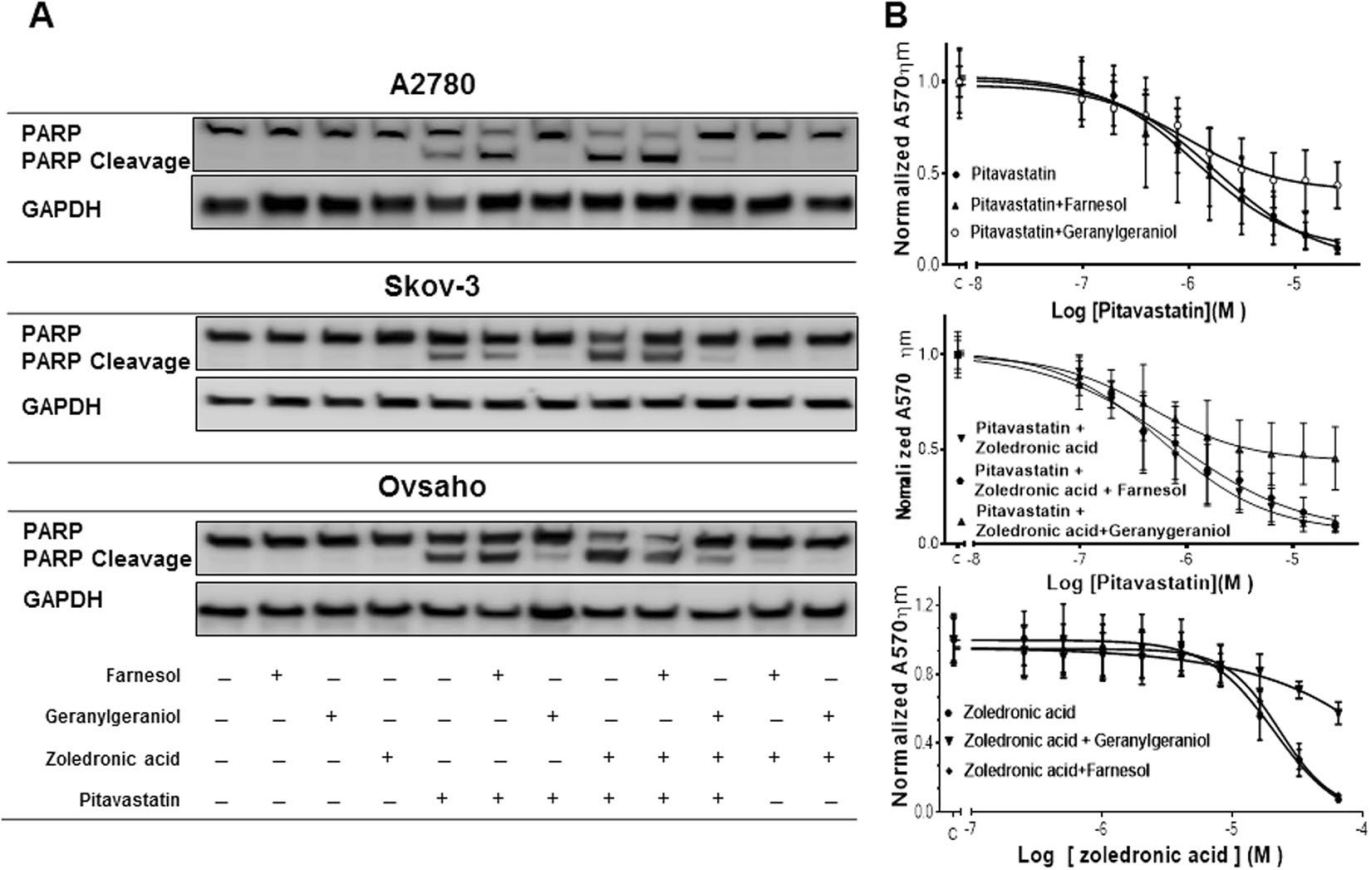
The effect of the pitavastatin-zoledronic acid drug combination is blocked by geranylgeraniol. (**A**) Cells treated with pitavastatin (A2780 and Ovsaho 1 µM and Skov-3 5 µM) and farnesol (10 µM) geranylgeraniol (10 µM) zoledronic acid (10 µM) for 48 hrs. PARP cleavage was assessed by immunoblotting. The results are representative of 3 experiments. (**B**) Skov-3 cell line was treated with pitavastatin or pitavastatin and zoledronic acid (10 µM) and geranylgeraniol (10 µM) and farnesol (10 µM) and after 72 hrs relative cell number was determined by staining with SRB. (**C**) morphological changes of Skov-3 cell line treated with indicated drug concentration for 72 hr were visualized by phase - contrast light microscopy.

To confirm that the reduction in cell viability and growth is attributable to apoptosis we assessed the effects of drugs alone and in combination on caspase activity and PARP cleavage. The combination of zoledronic acid and pitavastatin caused activation of the effector caspases 3/7 as well as caspase-8 (extrinsic pathway) and caspase-9 (intrinsic pathway). In all three cases, the caspase activation caused by the combination was significantly higher than that caused by pitavastatin alone (Fig. 3A,B and C). Subsequently, immunoblot analysis demonstrated that the combination resulted in accumulation of cleaved PARP that was greater than that observed with each single agent (Fig. 3C). To demonstrate that the effects of the drug combination were mediated by inhibition of HMGCR, we made use of the observation that mevalonate pathway metabolites can suppress the cytotoxic activity of statins^17, 32^. Importantly, the addition of geranylgeraniol, but not farnesol, blocked the cleavage of PARP induced by pitavastatin or the combination (Fig. 4A and B). As well as confirming that the combination works through inhibition of the mevalonate pathway, this also suggests that geranylgeranyl transferases play a more critical role than farnesyl transferases in the activity of pitavastatin. Finally, phase contrast microscopy revealed more pronounced rounding, blebbing or detachment from the plate in cells treated with the drug combination than in cells treated with the single agents (Supplementary Figure 1).

### The effect of pitavastatin and zoledronic acid on mevalonate pathway enzymes and p53

**Figure 5:**
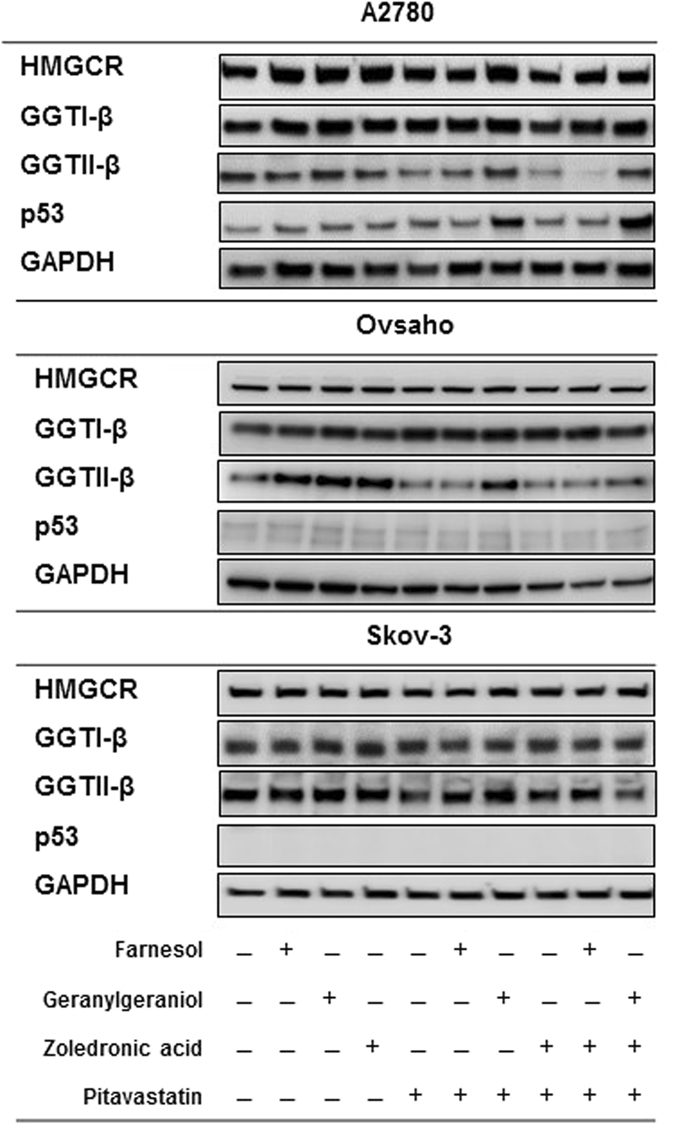
The effect of pitavastatin and pitavastatin-zoledronic acid on geranylgeranyl transferases. A2780, Skov-3 and Ovsaho cell lines were exposed to pitavastatin (1 µM, 5 µM and 1 µM, respectively) and zoledronic acid (10 µM) with and without geranylgeraniol (10 µM) and farnesol (10 µM) for 48 hrs. The levels of HMGCR, GGT-Iβ, GGT-IIβ and p53 were measured by immunoblotting of whole cell lysate. GAPDH was used as a loading control. The results are representative of 3 experiments.

To explore further the mechanism of the drug combination, we assessed the effect of the drug combination on mevalonate pathway enzymes. Pitavastatin decreased the level of GGT-IIβ without a significant effect on HMGCR, GGT-Iβ and p53 levels in A2780, Skov-3 (p53 null) and Ovsaho cells. This was also blocked by the inclusion of geranylgeraniol but not farnesol (Fig. 5). The combination of pitavastatin and zoledronic acid also reduced the level of GGT-IIβ and this was also ameliorated by the inclusion of geranylgeraniol.

### Potentiation of pitavastatin activity requires inhibition of both GGT-Iβ and GGT-IIβ

**Figure 6:**
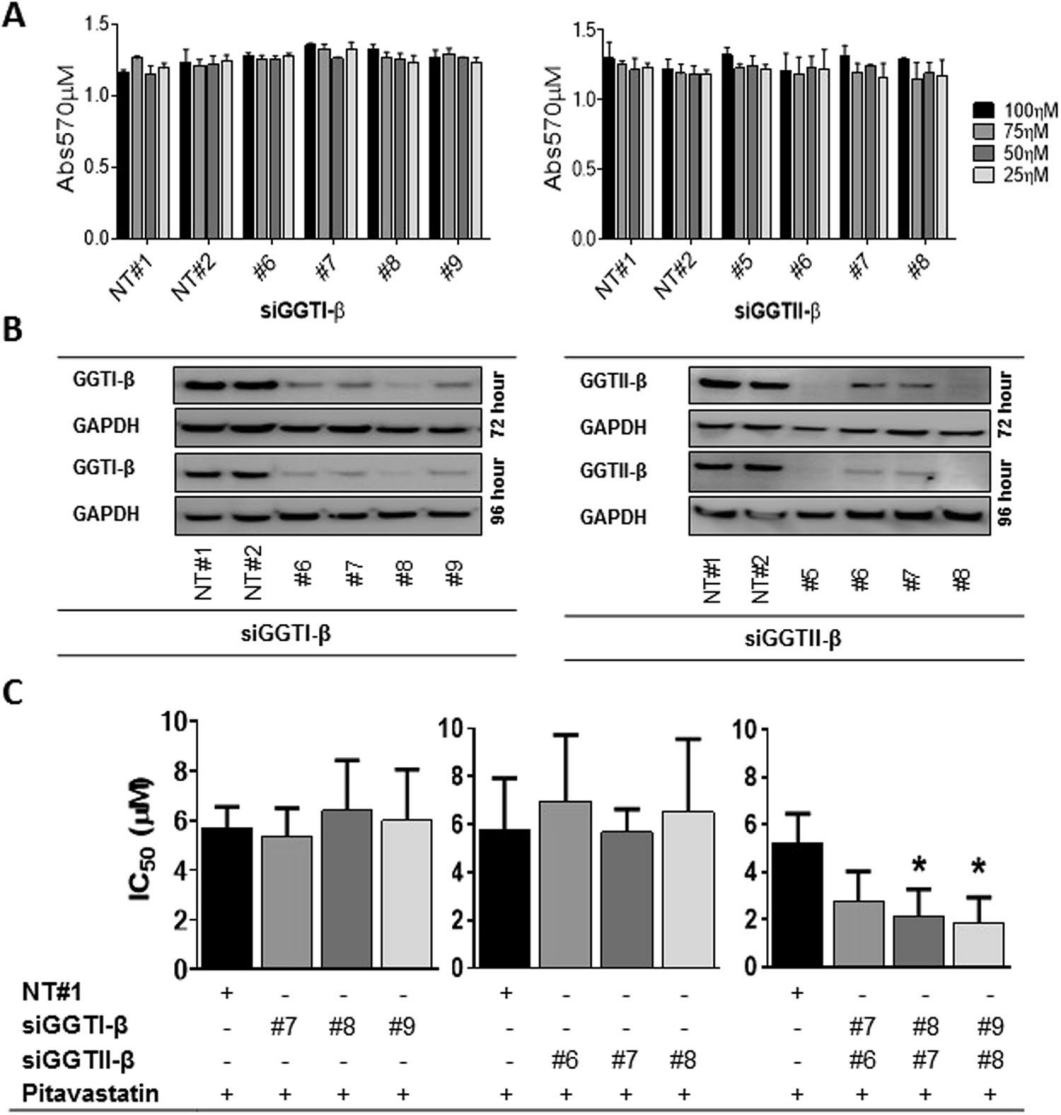
The effect of knockdown of GGT-Iβ and GGT-IIβ on sensitivity to pitavastatin. (**A**) Ovcar-4 cells exposed to 3 separate siRNA to GGT-Iβ and GGT-IIβ did not affect cell growth assessed by staining with SRB. (**B**) Ovcar-4 cells were transfected with the indicated siRNA and GGT-Iβ and GGT-IIβ measured by immunoblotting after 72 and 96 hrs. (**C**) Ovcar-4 cells were transfected with siRNA and after 24 hrs exposed to pitavastatin for a further 72 hrs before cell number was estimated by staining with SRB. The IC_50_ of pitavastatin in combination with siRNA of GGT-Iβ and GGT-IIβ (mean ± SD, n = 3) was significantly different from cells transfected with non-targeting siRNA (NT#1) where shown (*P < 0.05, one-way ANOVA followed by Tukey’s post-hoc test).

**Figure 7:**
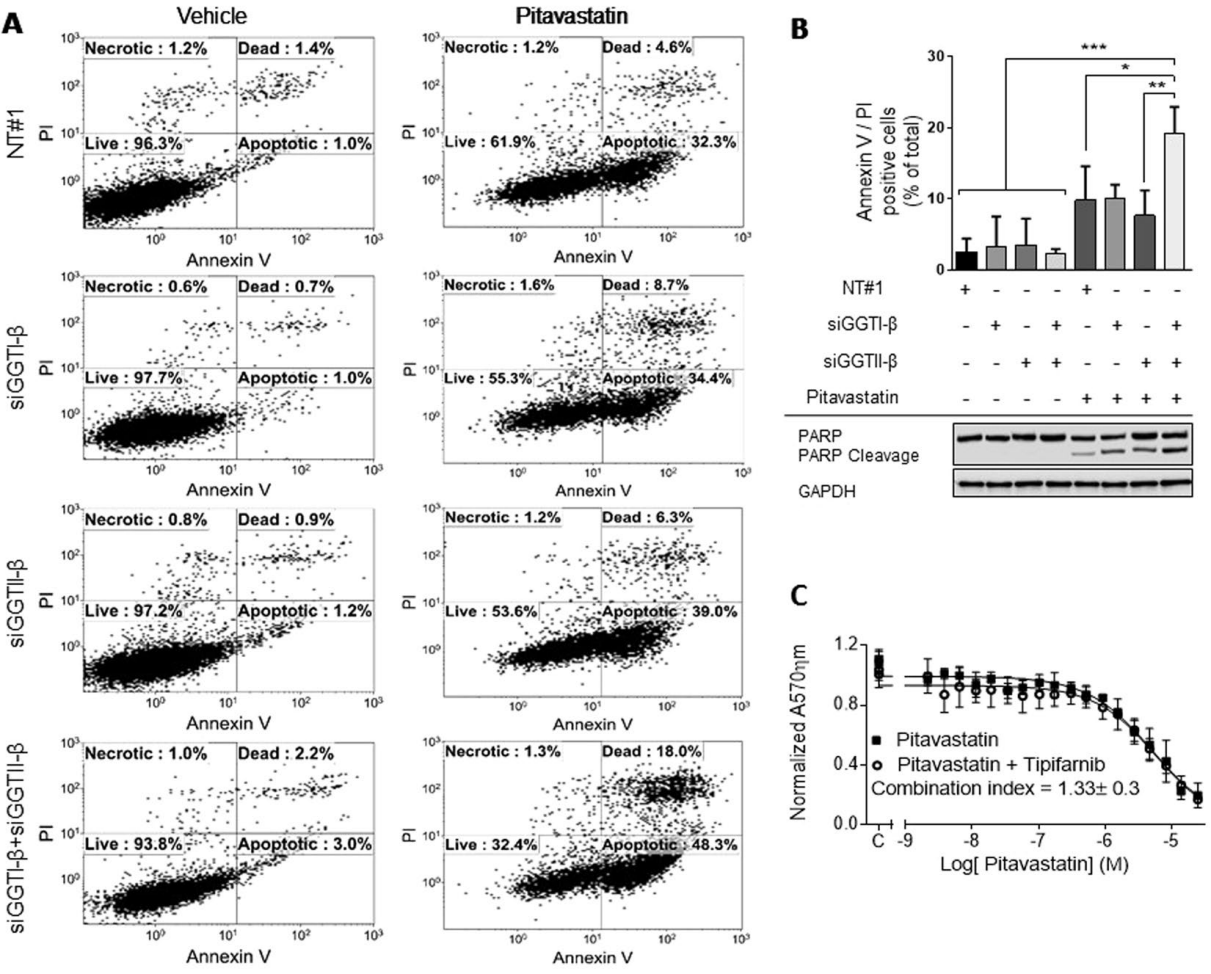
The effect of pitavastatin and pitavastatin–siGGT-Iβ and siGGT-IIβ combinations on apoptosis. (**A**). Ovcar-4 cells were transfected with siRNA to GGT-Iβ and GGT-IIβ and exposed to pitavastatin (10 µM) for 48 hr. After labelling with annexin V/propidium iodide the cells were analysed by flow cytometry. (**B**) The annexin V and propidium iodide positive cells were quantified (mean ± SD, n = 3) and were significantly different from cells transfected with non-targeting siRNA where shown (*P < 0.05; **P < 0.01; ***P < 0.001; one-way ANOVA followed by Tukey’s post-hoc test). In parallel, PARP cleavage determined by western blotting. (**C**) The activity of pitavastatin in a cell growth assays were measured in the absence and presence of tipifarnib (0.25 µM) and combination index calculated.

To help understand the mechanism of the drug combination in more detail, we investigated further the mechanism of action of pitavastatin. To evaluate whether the cytotoxic activity of pitavastatin relies upon inhibition of geranylgeranylation of specific proteins or the family of isoprenylated proteins in general, we set out to identify whether substrates of GGT-I or GGT-II were critical to the activity of pitavastatin. We hypothesized that if inhibition of prenylation by one or both of these geranylgeraniol transferases was essential for the cytotoxic activity of pitavastatin, then siRNA knockdown of one of them should increase the potency of pitavastatin and that this would facilitate subsequent identification of the proteins whose geranylgeranylation is critically affected by pitavastatin. For these studies, we used Ovcar-4 cells because recent data has suggested they are more representative of high grade serous ovarian carcinoma than A2780 or Skov-3 cells^35^. We tested the effect of knockdown of GGT-Iβ and GGT-IIβ using siRNA at concentration which inhibited the expression of these enzymes without causing significantly reduction in cell viability (Fig. 6A and B). Knockdown of either GGT-Iβ or GGT-IIβ alone using 3 separate siRNA did not significantly increase the potency of pitavastatin_._ However, inhibition of both GGT-Iβ and GGT-IIβ simultaneously using 3 separate siRNA combinations resulted in a significant increase in sensitivity to pitavastatin, shown by a significant decrease in pitavastatin IC_50_ compared to control cells exposed to non-targeting siRNA (Fig. 6C). In confirmation of this, combined knockdown of both geranylgeranyl transferases and exposure to pitavastatin led to significantly more Annexin V/PI labelling (Fig. 7A) and more PARP cleavage (Fig. 7B) compared to treatment of cells with pitavastatin alone. In contrast, inhibition of farnesyl transferase with tipifarnib did not augment the activity of pitavastatin and an additive interaction was observed (Fig. 7C).

### Pitavastatin and pitavastatin-zoledronic acid alter the subcellular localization of small GTPases

**Figure 8:**
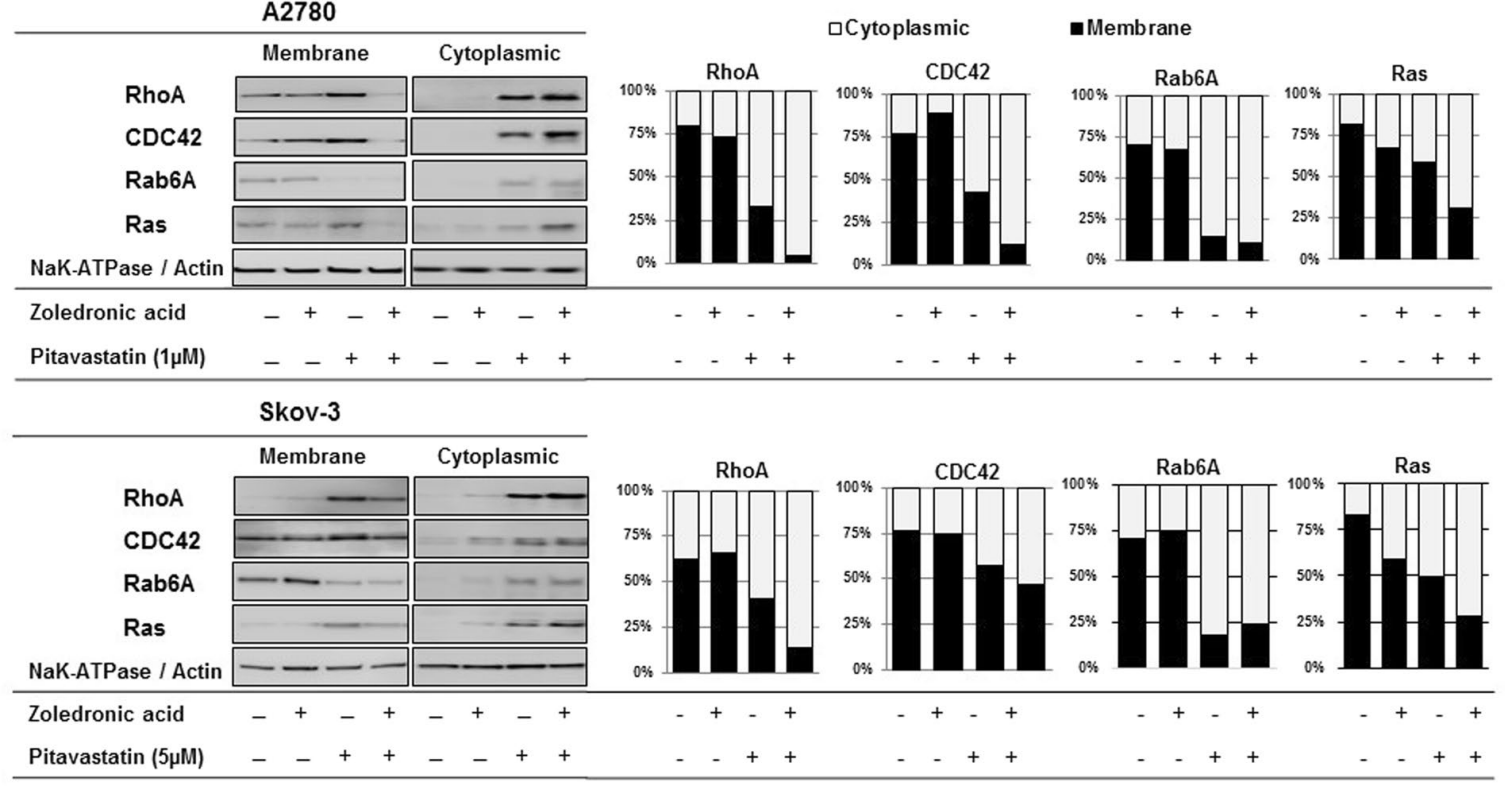
The effect of pitavastatin and pitavastatin-zoledronic acid on the subcellular localization of small GTPases. Lysates of A2780 and Skov-3 cells that had been treated with indicated drugs for 48 hr were fractionated into cytoplasm and membrane and analyzed by immunoblotting. The graphs show the mean fraction recovered in the cytosolic or membrane fractions (n = 3).

These data suggested that blocking geranylgeranylation may be crucial to the cytotoxic activity of drug combinations involving pitavastatin. Attachment of geranylgeraniol to small GTPases is necessary for their membrane localization. This led us to assess the effect of the drug combination on the subcellular localization of small GTPases as an indication of the impact of the drugs on the mevalonate pathway (Fig. 8). Cells were treated with pitavastatin and/or zoledronic acid, the cells fractionated into cytoplasmic and membrane fractions and the distribution of RhoA, CDC42, Rab6A and Ras was examined. Although zoledronic acid used as a single agent did not affect the membrane localization of these small GTPases, pitavastatin decreased the proportion of RhoA, CDC42, Rab6A and Ras proteins found in the membrane fraction and also caused a reciprocal increase in the cytosolic fraction. When cells were treated with pitavastatin and zoledronic acid, the loss of small GTPases from the membrane fraction to the cytosolic fraction was augmented.

## Discussion

We have previously established that pitavastatin is the statin most likely to be effective in the treatment of cancer^17, 33^. Although repurposing statins for use in oncology is attractive, there are legitimate concerns about the potential for myopathy^36^ and this makes it desirable to identify drugs which could potentially reduce the dose of pitavastatin administered to patients. In this study, we have found zoledronic acid acts synergistically with pitavastatin using several different assays and in several different cell lines. The drug combination was synergistic when assessed in two cell growth assays, a cell survival assay and also in several assays measuring apoptosis. This is significant because zoledronic acid is a drug in clinical use and so may reasonably be combined with pitavastatin in the treatment of cancer patients. Pitavastatin inhibits HMGCR, reducing the supply of mevalonate that is used to synthesize isoprenoids and zoledronic acid is known to inhibit farnesyl diphosphate synthase^37–40^ which is also part of the mevalonate pathway. Previous studies have identified synergy between statins and zoledronic acid^21, 41–44^. In this study, we further explored the mechanism underlying synergy between pitavastatin and zoledronic acid. In particular, we found that pitavastatin reduces levels of GGT-IIβ. This is significant because it demonstrates that the drug combination inhibits the mevalonate pathway at three different points. The drug combination also profoundly reduces the amount of small GTPases associated with the cell membrane, suggesting a potential mechanism by which the drugs trigger apoptosis. We also found that simultaneous inhibition of both GGT-I and GGT-II, but not of either transferase individually, potentiates the activity of pitavastatin. This has significant implications for understanding the mechanism by which pitavastatin induces cell death and for drug discovery programmes to identify compounds which inhibit geranylgeranyltransferases synergistically with pitavastatin.

Our results, and several previous studies, have suggested that the cytotoxic effect of statins in cancer cells result from inhibiting the synthesis of geranylgeraniol^12, 32, 45^. Indeed, the importance of this isoprenoid in oncogenesis is underlined by the observation that geranylgeraniol promotes tumor growth in xenograft-bearing mice^46^. In this study, we confirmed that geranylgeraniol is able to inhibit the cytotoxic effects of the pitavastatin-zoledronic acid drug combination in wild-type *TP53* (A2780 cells), mutated *TP53* (Ovsaho) and cells lacking *TP53* (Skov-3). This observation is significant because we have proposed that relatively high doses of statins will be necessary to treat cancer to provide an adequate plasma concentration (microMolar) of the drug in patients, leading to the concern that high concentrations of pitavastatin might be cytotoxic through a mechanism other than inhibition of HMGCR. Our data provides several other lines of evidence in support of pitavastatin exerting its effect through inhibition of HMGCR. Firstly, the observation that geranylgeraniol, a product of the mevalonate pathway, suppresses the effects of pitavastatin support pitavastatin working through an “on-target” mechanism. Secondly, our observation of synergy between two sets of drugs inhibiting the same pathway (pitavastatin and bisphosphonates) is also consistent with the effect of pitavastatin being mediated by HMGCR. Finally, we also found that siRNA directed to geranylgeranyl transferases, a part of the mevalonate pathway, potentiated the activity of pitavastatin. In summary, the synergy between pitavastatin and several reagents targeting the mevalonate pathway strongly supports the argument that pitavastatin, even at microMolar concentrations, acts principally through inhibition of HMGCR and the mevalonate pathway. This conclusion is of crucial importance to the design of clinical trials, because understanding the mechanism of action of pitavastatin in cancer is essential for selecting which patients should receive the drug.

The suppression of the activity of pitavastatin-zoledronic acid combinations by geranylgeraniol suggested that inhibition of the production of this isoprenoid was central to the effect of the drug combination. However, this observation did not indicate whether the effect of pitavastatin reflects inhibition of geranylgeranylation of a crucial subset of proteins or whether inhibition of protein prenylation more broadly underlies the effect of pitavastatin. This is not a trivial issue to tackle because around 2% of mammalian proteins undergo post-translational prenylation^47^. Athough Ras superfamily GTPases are obvious candidates affected by pitavastatin, the sensitivity of multiple myeloma cells to lovastatin was not modulated by ectopic expression of individual constitutively active Ras, RhoA, RhoB, Rac1, and Cdc42 small GTPase proteins^48^. To begin to address this, we first considered which geranylgeranyl transferases might be most significantly affected by pitavastatin. We hypothesized that if the effects of pitavastatin were mediated by preventing the prenylation of a substrate of either GGT-Iβ or GGT-IIβ, then synergy would be observed between pitavastatin and siRNA to one of these geranylgeraniol transferases. We anticipated using this information to focus on substrates for one transferase in our search for the proteins whose geranylgeranylation is affected by pitavastatin and pitavastatin-zoledronate combinations and which is necessary for the cytotoxic activity of these drugs. However, we found that siRNA to either one of the transferase alone was insufficient to potentiate the activity of pitavastatin in both cell growth assays and in two apoptosis assays. However, when we combined different siRNA to simultaneously repress both geranylgeraniol transferase I and II, the potency of pitavastatin was increased. We observed this using three separate siRNA combinations. In contrast, inhibiting farnesyltransferase by tipifarnib was not synergistic with pitavastatin. This confounded our approach to understanding the mechanism of action of pitavastatin and pitavastatin/zoledronate because these results did not implicate one single geranylgeranyl transferase. Instead, these data suggest that pitavastatin exerts its cytotoxic activity by preventing the geranylgeranylation of several proteins whose prenylation is catalysed by GGT-Iβ and/or GGT-IIβ. It is likely that these same proteins are affected by the pitavastatin-zoledronic acid combination. We cannot rule out, however, that the activity of the pitavastatin-zoledronic acid combination depends on blocking the prenylation of a small subset of unidentified proteins that can be redundantly isoprenylated by either GGT-Iβ or GGT-IIβ. Redundancy between these prenyl transferases explains why inhibition of both GGT-Iβ and GGT-IIβ was found to be necessary for synergy with pitavastatin because one transferase can compensate for the depletion of the other. The idea of redundancy between the transferases is plausible because these enzymes do not exhibit absolutely inflexible substrate specificity and geranylgeranylation has even been reported as a mechanism of resistance to farnesyl transferase inhibitors^49^. The apparent redundancy observed between GGT-I and GGT-II also provides important information for drug discovery programmes designed to identify compounds which are synergistic with pitavastatin. The data suggests that targeting selectively either GGT-I or GGT-II may be futile because one transferase may compensate for inhibition of the other. Compounds which inhibit both transferases may be necessary. Indeed, we did not see synergy when we combined pitavastatin with GGTI-2133 which inhibits GGT-Iβ but not GGT-IIβ. Rather, GGTI-2133 was antagonistic with pitavastatin, although this may reflect off-target effects of this compound^50, 51^.

To confirm that pitavastatin, zoledronic acid and the combination of the two drugs resulted in altered protein prenylation, we measured the effect of these drugs on several small GTPases. We selected these as relevant targets affected by pitavastatin because small GTPases proteins are well known to be prenylated and are involved in regulation of several signalling pathways involved in cell growth and survival^52^. Pathways known to be regulated by small GTPases include the PI3K/AKT and Raf/Mek/MAPK/ERK pathways which regulate cell cycle progression and apoptosis^14^. We selected substrates of GGT-Iβ (RhoA, CDC42) or GGT-IIβ (Rab6A) as well as of farnesyltransferase (Ras)^49^ to evaluate the effect of the pitavastatin-zoledronic acid combination. Pitavastatin increased the proportion of all four small GTPases that was found in the cytosolic fraction, consistent with inhibition of prenylation. In both cells lines pitavastatin also increased the amount of RhoA, CDC42 and Ras found in the cell membrane, suggesting that loss of prenylation may lead to an increase in the abundance of these small GTPases. Upregulation of Ras and Rho by statins has been observed previously^42, 53^ as a result of increase translation or reduced turnover^54^. In contrast, there appeared to be a reduction in the total amount of Rab6A, consistent with our previous results^33^. The combination of zoledronic acid with pitavastatin increased in most cases the proportion of small GTPases found in the cytosolic fraction. Taken together, our data suggests that the synergy between pitavastatin and zoledronic acid inhibits the mevalonate pathway at multiple points and leads to a profound reduction in the membrane localization of small GTPases. Since several of these GTPases regulate cell survival and proliferation, the loss of membrane localization of these proteins is likely to contribute to the synergistic inhibition of cell growth and survival. We cannot rule out, however the possibility that the cytosolic form of these proteins inhibits cell growth and survival^55^.

We observed that pitavastatin, alone and in combination with zoledronic acid, decreases the level of GGT-IIβ. Thus, the pitavastatin-zoledronic acid drug combination inhibits at least three points on one biosynthetic pathway and it is likely that this contributes to the synergy which we have observed in almost all the cell lines we tested. This is also significant because it suggests that reduced GGT-IIβ is likely to contribute to the activity of these drugs, although the mechanism underlying the reduction in GGT-IIβ is not yet clear. However, mevalonate pathway enzymes are regulated by feedback and feedforward mechanisms^56, 57^. The reduced supply of geranylgeraniol may cause changes in the level of the enzyme for which it is a substrate. This observation raises the possibility that pitavastatin may be particularly useful in cancers in which GGT-IIβ is either abundantly expressed or mutated such as ovarian cancer^11, 58^. In addition, overexpression of GGT-II enzyme substrates such as Rab25, Rab5 and Rab7, has been reported in breast, ovarian, prostate and bladder cancers, and for some of these substrate mutation is a determinant of the aggressiveness of the cancer and a predictor of poor outcome^59^.

A number of issues remain to be addressed. Although zoledronic acid was synergistic with pitavastatin in the majority of cell lines, the drug combination was antagonistic in Ovcar-3 cells. It is also unclear why we observed less synergy when pitavastatin was combined with risedronate instead of zoledronic acid. Indeed, an antagonistic interaction as observed between risedronate and pitavastatin in Ovcar-3 cells as well as Ovcar-8 cells. We can currently only speculate on the cause for these observations. In the case of Ovcar-3 cells the presence of insulin in the Ovcar-3 growth medium, but not in the media for other cell lines, may contribute. The genetic background of the cells is also likely to play a key factor, but the identification of additional cell lines in which antagonism is observed would be necessary to assist in identifying mutations or epigenetic changes which are associated with antagonism between bisphosphonates and statins. We also do not yet have a clear model of the link between reduced protein prenylation and the induction of apoptosis. We observed activation of both caspase 8 and caspase 9, as well as the effector caspases 3/7. This may represent separate activation of both the extrinsic and intrinsic pathways or cross-talk between these pathways, for example by cleavage of BID. Further studies are required to address these issues.

We conclude that inhibition of farnesyl diphosphate synthase by zoledronic acid offers a promising strategy to increase the efficacy of statins in cancer patients. Statins and bisphosphonates generally have a good safety profile and are available clinically in relatively cost-effective generic forms^56, 60, 61^, making this approach particularly attractive. The inclusion of zoledronic acid alongside pitavastatin in clinical trials of patients with ovarian cancer warrants urgent consideration. In particular, these trials will need to evaluate whether the inclusion of zoledronic acid potentiates the efficacy of pitavastatin without an increased risk of myopathy which is associated with statin use.

## Material and Methods

### Compounds

Pitavastatin (Livalo, Adooq), zoledronic acid and risedronate (Selleck), and GGTI-2133, Tipifarnib, farnesol, geranylgeraniol and mevalonate (Sigma-Aldrich) were prepared as 20 mM solutions in DMSO except zoledronic acid which was dissolved in H_2_O.

### Cell culture

A panel of ovarian cancer and normal cell lines (HOE, A2780, CisA2780, Cov-318, Cov-362, Ovcar-3, Ovcar-4, Ovcar-5, Ovcar-8, Ovsaho, Igrov-1 and Skov-3) were incubated in a humidified incubator at 37 °C in 5% CO_2_ atmosphere. Cell lines, were maintained in RMPI-1640 medium with the exception of Cov-318 and Cov-362 cell lines were grown in DMEM. The media of all cell lines were supplemented with 10% fetal bovine serum, 2mM L-Glutamine and 50IU/ml of penicillin/streptomycin. Additionally, Ovcar-3 medium was supplemented with 0.01 mg/ml bovine insulin and 1 mM sodium pyruvate.

### Cell growth assay

Human ovarian cells were subcultured in 96-well plates (5000 cells/well except A2780, CisA2780 and Ovcar-8 for which 2500 cell/well were seeded) overnight before incubation with the indicated drugs. Cells were exposed to serial dilutions of individual drugs for 72 hr, except for the Cov-318 and Cov-362 cells which, due to their slow rate of growth (doubling time 76 hr and 110 hr respectively), were incubated for 120 hr. Relative cell number was estimated by staining with sulforhodamine B as previously described^62^. Complete concentration–response curves were determined in every experiment, using serial drug concentrations to establish IC_50_ values and Hill coefficients. Graphpad Prism was used to analyse the data to fit a four-parameter Hill equation using non-linear regression. For drug combinations, fixed dose (non-fixed ratio) drug combinations^34^ were used in which a fixed concentration of zoledronic acid (10 µM), risedronate (10 µM) or GGTI-2133 (5 µM) were combined with a serial dilution of pitavastatin. Combination indices were calculated as described (Chou &Talalay) at fraction affected = 0.5﻿﻿^63^. In some experiments, cells were also exposed to 10 μM farnesol, or 10 μM geranylgeraniol.

### Cell Titer-Glo Luminescent Assay (ATP-assay)

Cell growth assays were prepared as described above but instead of staining with SRB, intracellular ATP level was quantitated using the cell Titer-Glo Luminescent assay reagent (Promega, Madison, WI, USA). The Bliss independence^64^ criterion was calculated to determine the expected effect of the drug concentration and this was compared the observed effect of the combination.

### Trypan Blue Assay

Cells (2 × 10^5^/well/2 ml) were seeded per well of a 6 well plate. The next day, the indicated drugs were added. After 72 hr, adherent cells were collected by trypsinization and combined with the non-adherent floating cells. The cells were centrifuged at 150 g for 3 minutes, the pellets were re-suspended gently in medium and stained by 0.2% Trypan Blue. The viable and non-viable cells were counted with a haemocytometer, and the effect of the combination compared to that expected from the Bliss independence criterion.

### Caspase-Glo 3/7, 8 and 9 Assays

For caspase assay, 5000 cells were plated in 80 µl of medium in 96 well plates. Two plates were prepared for each experimental condition. After 48 hrs, caspase activity was measured using Caspase-Glo 3/7, 8 or 9 reagent (Promega, Madison, WI, USA) by adding 20 µL of detection reagent. Caspase activity was measured after half hour incubation of cells with reagent, using a microplate reader. The second plate was stained with SRB and the caspase activity was normalised to the SRB stain. The effect of the combination was compared to that expected from the Bliss independence criterion.

### Total and fractionated protein separation

To prepare cell lysates or cell fractions, A2780 or Skov-3 cell line (2 × 10^5^/well/2 ml) were seeded in 6 well plates. After 48 hr incubation with drugs alone and in combinations, cells were collected. Cell lysates were prepared as described^65^ and protein concentration measured by BCA assay. Equal masses of the sample proteins were separated by electrophoresis and transferred to a PVDF membrane. The membrane was incubated overnight at 4 °C with primary antibodies: anti-PARP (1:1000) (#95425; Cell Signaling Technology); anti-HMGCR (1/1000) (ab174830; Abcam); anti-GGT-Iβ subunit (1/1000) (sc3765901; Santa cruz); anti-GGT-IIβ subunit (1/1000) (sc376854; Santa cruz); anti-p53 (1/5000) (ab179477; Abcam); anti-actin (1/1000) (#4968; Cell Signal Technology) or with anti-GAPDH antibody (1:5000) (mab374; Millipore) as loading control. Proteins were visualised using peroxidase-conjugated secondary antibodies and Uptilight™ Ultra WB Chemiluminescent Substrate (Interchim, France).

For cytoplasmic and membrane protein separation, cells were seeded as described above. Membrane and cytoplasm proteins were separated using Mem-PER™ Plus Membrane Protein Extraction Kit (Thermo-scientific) according to the manufacturer’s protocol. Cell pellets were first incubated for 10 minutes with 150 µl permeabilization buffer at 4 °C with constant mixing. Permeabilized cells were centrifuged for 15 min at 16000 × *g* and the supernatant which contained the cytosolic protein were collect carefully and transferred to new tube. 0.1 mL of solubilization buffer was used to suspend pellets. The suspensions were incubated at 4 °C for 30 minutes with constant mixing. Tubes were centrifuged at 16,000 × *g* for 15 minutes at 4 °C and the supernatant containing the solubilized membrane and membrane-associated proteins were transferred to a new tube. Both the cytoplasmic and membrane fraction were quantitated by BCA assay and stored at −80 °C. These samples were separated by electrophoresis as described above and analysed with by immunoblotting: Anti-RhoA (1/1000) (ab187027; Abcam), Anti-CDC42 (1/1000), Anti-Rab6A (1/1000)(ab95954; Abcam) and Anti-Ras (1/1000) (ab52939; Abcam). Na/K-ATPase (1:10000) (ab76020; Abcam) used as loading control for the membrane fraction.

### siRNA of the Geranylgeranyl transferase I-β (GGT-Iβ) and Geranylgeranyl transferase II-β (GGT-IIβ)

5000 cells/well in 96 well plate or 1 × 10^6^ cells/well in 12 well plate, were transfected with 100 nM of an GGT-Iβ#6, #7, #8, #9 or GGT-IIβ#5, #6, #7, #8 or non-targeting#1(NT#1) (Dharmacon) using Dhamafect-1 as described previously^62^. The next day, the cells were exposed to a 18 serial dilutions of pitavastatin. After a further 72 hrs, the cells were stained with SRB. Knockdown was confirmed by western blotting using GGT-Iβ and GGT-IIβ antibodies.

### Flow cytometry

The Annexin VFITC kit (Miltenyi biotech) was used to evaluate the effect of siRNA combinations with pitavastatin on Ovcar-4 cell line using flow cytometry according to the manufacturer’s instructions. Ovcar-4 cells were seeded at a density of 1 × 10^5^ cell per well in 12-well plates overnight. The cells were exposed to pitavastatin for 48 hr after 24 hr incubation with transfected GGT-Iβ, GGT-IIβ and NT#1 oligos. Cells were trypsinized and washed in ice-cold PBS and centrifuged at 300 *g* for 5 minutes to form pellets. The pellets were re-suspended in 1 ml of binding buffer and centrifuged for 10 minutes at 300 g. The pellets were re-suspended in 100 µl of annexin V binding buffer and 10 µl of Annexin V fluorochrome were added to each sample and incubated for 10 minutes in the dark at room temperature. The washing step were repeated with 1 ml of annexin V binding buffer. Lastly, the cells re-suspended in 500 µl Annexin V Binding Buffer and 5 µl of propidium iodide (1 µg/ml) were added before the analysis by flow cytometry. The viability of cells was defined as live (annexin V-negative and PI-negative), apoptotic cells (annexin V-positive and PI-negative), dead cells (annexin V-positive and PI-positive) and necrotic cells (annexin V-negative and PI-positive).

## Electronic supplementary material

Supplementary info
