# Supplementary material for: Inhibition of the mevalonate pathway augments the activity of pitavastatin against ovarian cancer cells
Source: Sci Rep. 2017 Aug 14;7:8090. doi: 10.1038/s41598-017-08649-9 (PMC5556066; doi:10.1038/s41598-017-08649-9)
Supplement: Supplementary file 1 — Supplementary info [file 41598_2017_8649_MOESM1_ESM.pdf]

Supplementary Information

Inhibition of the mevalonate pathway augments the activity of pitavastatin against ovarian cancer cells

Marwan Ibrahim Abdullah, Mohammed Najim Abed and Alan Richardson

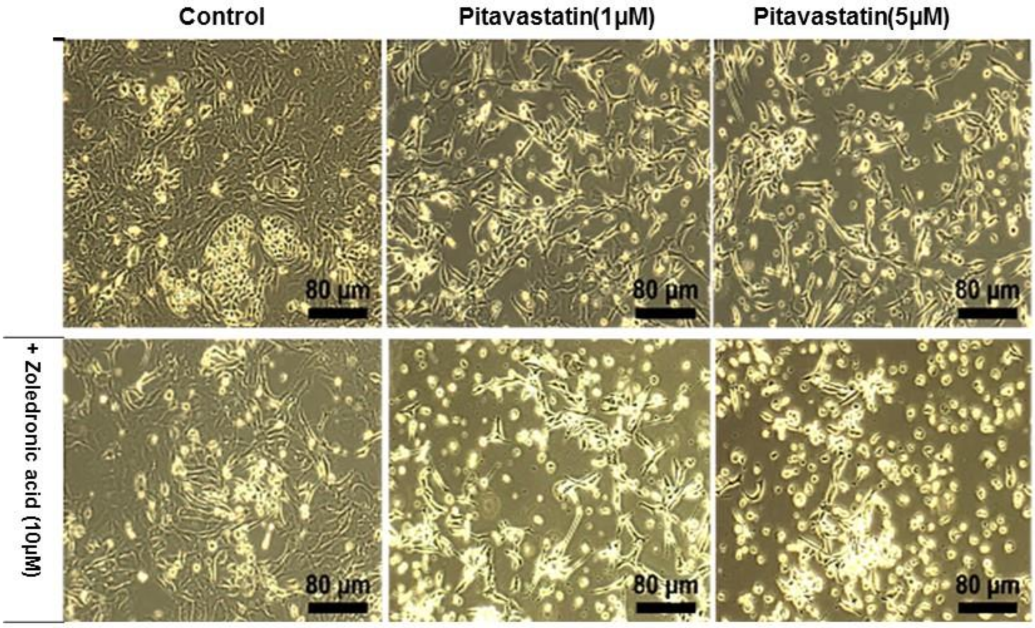

Supplementary Figure 1. Vsualization of synergy between pitavastatin and zoledronic acid by phase contrast microscopy. Skov-3 cell line treated with indicated drug concentration for 72 hr were visualized by phase contrast light microscopy.
